# Cognitive Reserve, Leisure Activity, and Neuropsychological Profile in the Early Stage of Cognitive Decline

**DOI:** 10.3389/fnagi.2020.590607

**Published:** 2020-10-26

**Authors:** Sook Young Lee, Jae Myeong Kang, Da Jeong Kim, Soo Kyun Woo, Jun-Young Lee, Seong-Jin Cho

**Affiliations:** ^1^Department of Psychiatry, Gil Medical Center, Gachon University College of Medicine, Incheon, South Korea; ^2^Department of Neuropsychiatry, SMG-SNU Boramae Medical Center, Seoul National University College of Medicine, Seoul, South Korea

**Keywords:** cognitive reserve, neuropsychological tests, leisure activity, cognition, mild cognitive impairment

## Abstract

In older adults with normal cognition, cognitive reserve (CR) is known to be associated with the neuropsychological profile. We investigated the association between comprehensive CR and detailed neuropsychological profile in the early stage of cognitive decline. Fifty-five participants with mild cognitive impairment or subjective cognitive decline completed the cognitive reserve index questionnaire (CRIq) that yielded total, education, working activity, and leisure time scores (CRI-Total, CRI-Education, CRI-Working activity, and CRI-Leisure time, respectively). Mini-mental state examination (MMSE) and detailed neuropsychological evaluation were performed. Psychiatric symptom scales were applied to measure depression, apathy, positive or negative affect, and quality of life. Correlation and linear regression analyses of the variables were performed. The effect of CR-Education, CRI-Working activity, and CRI-Leisure time on the composite cognitive score was determined using a multivariable regression model. We observed that for CRI-Total (*B* = 3.00, *p* = 0.005), CRI-Education (*B* = 3.39, *p* = 0.002), and CRI-Leisure time (*B* = 2.56, *p* = 0.015), CR correlated with MMSE scores, while only CRI-Leisure time associated with the naming ability (*B* = 2.20, *p* = 0.033) in the detailed neuropsychological test results of the participants. Multivariable regression model also indicated that among CRI subscores, CRI-Leisure time directly affects the composite cognitive score (β = 0.32, *p* = 0.011). We found that in the early stage of cognitive decline in older adults, comprehensive CR was associated with global cognition, and only leisure activity was identified to be associated with the detailed neuropsychological profile including naming ability. These results may imply the positive effect of leisure activity on cognitive function in the early stages of cognitive decline.

## Introduction

Aging and cognitive decline are major medical and social issues. The number of older adults will continue to grow worldwide, increasing the burden of aging (Pison, 2019). Approximately 10% of elderly people experience dementia, a common clinical syndrome often caused by underlying neurodegenerative diseases (Alzheimer’s Association, 2016). The burden of dementia increases because dementia is a syndrome deteriorating cognition, behavioral, and psychological symptoms, and activities of daily living (American Psychiatric Association, 2013). Alzheimer’s disease (AD), is the most prevalent form of dementia in elderly people, and to date, many efforts to develop effective treatments for AD have failed. Thus, preventing dementia is gradually gaining more support (Cummings et al., 2018).

It is known that the cognitive reserve (CR) can enhance the cognitive function against normal and pathologic aging (Stern, 2002; Scarmeas and Stern, 2004; Soldan et al., 2020). CR is a modifiable factor that can be changed or improved, and this is the basic theory behind cognitively, mentally, and physically stimulating activities to delay cognitive decline and dementia (Reed et al., 2010; Clare et al., 2017). CR has been measured by factors such as premorbid intelligence quotient (IQ), years of education, complexity of occupation, and composites of hobbies and leisure activities (Jones et al., 2011). It is well known that older adults with a higher level of education show better global and detailed neuropsychological function (Thow et al., 2017; Groot et al., 2018; Gu et al., 2018; Lavrencic et al., 2018; Zhang et al., 2019; Zarantonello et al., 2020) than those with lower education levels. Healthy lifestyle, including cognitive, social, and physical activities, has also been positively correlated with global cognition (Clare et al., 2017).

In terms of measurements, CR proxies such as education (MacPherson et al., 2017; Rodriguez et al., 2019; Zhang et al., 2019), IQ (Ghaffar et al., 2012; MacPherson et al., 2017), and occupational attainment (Ghaffar et al., 2012; Boots et al., 2015) have been used to represent a component of CR (Jones et al., 2011) rather than a comprehensive lifetime cognitive stimulating activity. In addition, previous studies have determined the effect of CR on cognitive function, as measured by global cognitive scales like the mini-mental state examination (MMSE) (Zhang et al., 2019) and the Montreal cognitive assessment (Park et al., 2019), and few have identified the relationship with comprehensive cognitive domains. Thus, an extended and thorough assessment of CR and a comprehensive neuropsychological evaluation may help to find their relationship. Additionally, most studies investigating CR and cognition were conducted in cognitively normal adults. Since educational and occupational attainment were found to affect the progression to dementia in patients with mild cognitive impairment (MCI) (Allegri et al., 2010; Myung et al., 2017), CR may play a protective role at the preclinical or prodromal stage of dementia that manifests with subtle decline of cognitive and psychiatric functions (Lyketsos et al., 2002; Robert et al., 2006).

We hypothesized that a higher comprehensive CR may correlate with a better detailed neuropsychological profile and psychiatric status in the early stage of cognitive decline. Hence, we assessed the association between comprehensive CR and a detailed neuropsychological profile and psychiatric symptoms, including depression, affect, and apathy.

## Materials and Methods

### Participants

From May 2019 to December 2019, individuals with subjective cognitive decline (SCD) or MCI were prospectively recruited from the Memory Clinic at Gil Medical Center, Gachon University. All participants complained of subjective cognitive impairment and were diagnosed with either SCD based on the clinical evaluation [clinical dementia rating-sum of boxes (CDR-SOB) ≤ 0.5] and neuropsychological test results (all cognitive domain *z*-score >-1.5 standard deviation), as previously described (Jessen et al., 2014; Molinuevo et al., 2017), or with MCI according to Petersen’s criteria (Petersen, 2004). The final diagnoses in the participants were confirmed by a board-certified psychiatrist (JM Kang). Patients with any of the following conditions were excluded: (1) Korean version of the MMSE score < 20; (2) CDR-SOB score >4.0; (3) impaired activities of daily living; (4) major psychiatric disorders; (5) history of diagnosis with any kind of dementia or cerebrovascular diseases; (6) severe medical or surgical comorbidities that may affect cognition such as cancer, chronic kidney disease, chronic obstructive pulmonary diseases, and the acute phase after any major surgery; and (7) history of neurodegenerative disorders, including Parkinson’s, Huntington’s, Pick’s, and Creutzfeldt-Jakob diseases. All participants provided written informed consent, and the Institutional Review Board of Gil Medical Center, Gachon University, approved the study (GAIRB2019-230).

### Assessments

All participants were evaluated for CR, comprehensive neuropsychological and clinical functions, and psychiatric symptoms. To evaluate CR, we used the cognitive reserve index questionnaire (CRIq) that was developed by Nucci et al. (2012) and validated in Korean normal adults (Choi et al., 2016). This questionnaire consists of 20 questions yielding scores for each of the following domains: years of education both formal and non-formal (CRI-Education); working activity (CRI-Working activity), which classifies working activities into five levels depending on the cognitive load required for the job involved and the number of years spent in each occupation; leisure time (CRI-Leisure time), to assess the type and frequency of cognitive activities such as reading books, attending concerts, and caring for pets the participants spend their free time on; and a total score (CRI-Total), with an average of 100 and a standard deviation of 15 for each score. Scores obtained for each domain were then adjusted for age.

Neuropsychological function was evaluated in all participants. For evaluation of the functional daily activity, CDR (score range of 0–3), CDR-SOB (score range of 0–15), and global deterioration scale (GDS; score range of 0–7) were also applied. The comprehensive neuropsychological tests consisted of subtests from the comprehensive neuropsychological test battery (Kang and Na, 2003). The digit span test and trail making test-A (TMT-A) were used to assess attention and the Seoul verbal learning test (SVLT) was used to assess verbal memory function (Kang and Na, 2003). The Rey-Osterrieth complex figure test (RCFT) copy test (Meyers and Meyers, 1995; Kang and Na, 2003) and the Korean version of the Boston naming test (K-BNT) (Kim and Na, 1999; Kang and Na, 2003) were used to assess visuospatial function and language ability, respectively. To evaluate the frontal executive functions, we used the TMT-B, controlled oral word association test (COWAT) animal, COWAT phonemic, and the Stroop test (color/word reading) (Kang and Na, 2003). Each neuropsychological test score was converted to a *z*-score based on its deviation from the overall score for the Korean elderly population with normal cognition of the same age and years of education. Additionally, a composite cognitive score and cognitive domain scores were calculated based on all the neuropsychological tests mentioned above that were validated for constructing a composite score in the Korean population (Jahng et al., 2015) by averaging standardized scores for each subtest (Bransby et al., 2019). All tests were evaluated by a board-certified neuropsychologist (SY Lee).

Psychiatric symptoms were also assessed. The geriatric depression scale (GDepS) was used to evaluate depressive symptoms. The GDepS is a validated, yes or no, 30-item questionnaire on mood, energy, anxiety, hopefulness, satisfaction, inattention, and insomnia, with higher scores indicating severe depression with a score range of 0–30 (Yesavage et al., 1982; Bae and Cho, 2004). The apathy evaluation scale (AES) was used to evaluate apathy. The AES is a validated, four-point Likert scale, 18-item questionnaire regarding apathy in the affective, behavioral, and cognitive aspects, with lower scores indicating severe apathy with a score range of 18–72 (Marin et al., 1991; Lee et al., 2013). The positive and negative affect schedule (PANAS) was used to evaluate affective symptoms. The PANAS is a validated, five-point Likert scale, 20-item questionnaire regarding alertness, enthusiasm, lethargy, and sadness (Watson et al., 1988; Lim et al., 2010). The PANAS yields scores for two domains, positive affect (PANAS-P) and negative affect (PANAS-N), with higher scores in PANAS-P indicating a higher positive affect with a score range of 10–50 and higher scores in PANAS-N indicating a higher negative affect with a score range of 10–50. The quality of life-Alzheimer’s disease (QOL-AD), validated in people with dementia, was used to evaluate the QOL of the participants. The QOL-AD consists of 13 items regarding physical health, friends, living situation, and ability to do things for fun, with higher scores indicating a better QOL with a score range of 13–52 (Thorgrimsen et al., 2003; Shin, 2006).

### Statistical Analyses

Demographic information and clinical data were analyzed with descriptive analysis. Correlations between CR and cognitive and psychiatric symptoms were analyzed using Pearson’s correlation coefficients in data with normal distribution and Spearman’s correlation coefficients in data with skewed distribution. Multiple comparisons were corrected by Benjamini–Hochberg false discovery rate method (Benjamini and Hochberg, 1995) in neuropsychological test results and psychiatric symptoms. Regression analyses were performed to determine the effect of CRIq scores on neuropsychological and psychiatric symptom test scores. To adjust covariates, sex and CDR-SOB or MMSE (for predicting CDR-SOB) were defined as independent variables as well as CRIq score, and each of the neuropsychological profiles or psychiatric symptoms were defined as a dependent variable. Since age and years of education were already adjusted as part of the neuropsychological test results and were included in the CRIq scores (Kang and Na, 2003; Choi et al., 2016), the dependent variables were only adjusted for sex. In the regression analyses, log transformation was performed for variables that did not distribute normally. The effect of each domain of CRIq on the composite cognitive score and subdomains of neuropsychological tests was determined using a path diagram of the multivariable regression model. A non-significant chi-square (χ^2^), a root-mean-square error of approximation (RMSEA) under 0.05, and a Comparative Fit Index (CFI) value above 0.95 indicated a strong model fit for the multivariable regression model, while a CFI above 0.90 and an RMSEA value under 0.08 indicated an adequate fit (Hu and Bentler, 1999; Kline, 2015). All analyses were performed with SPSS for Windows (SPSS, version 23; Chicago, IL, United States) except for the multivariable regression model, which was performed using AMOS for Windows (IBM SPSS, version 20; Chicago, IL, United States). *P* values < 0.05 (two-way) were considered significant.

## Results

### Demographic and Clinical Characteristics

Among the 58 participants who were enrolled in the study, two who failed to complete the assessments and one with a CDR-SOB score >4 were excluded from the analysis. Participants were then divided into two diagnostic groups: SCD (*n* = 36) and MCI (*n* = 19). Table 1 shows the demographics and the global cognitive scale scores for the participants. Mean age was 74.06 ± 6.43 in SCD and 75.42 ± 5.98 in MCI groups (*t* = -0.77, *p* = 0.446). Individuals with SCD were more likely to be male (χ^2^ = 4.52, *p* = 0.034) with significantly higher MMSE (*Z* = -2.53, *p* = 0.011) and lower CDR-SOB (χ^2^ = 18.41, *p* = 0.018) scores.

**TABLE 1:** Demographics and clinical characteristics of the participants.

		SCD (*n* = 36)		MCI (*n* = 19)		*t*, *Z*, or χ ^2^, *p*
		Mean ± SD	Min, max	Mean ± SD	Min, max	
		or number	or percent	or number	or percent	
Age, years^†^		74.06 ± 6.43	62, 90	75.42 ± 5.98	64, 82	-0.77, 0.446
Sex (female; *n*,%)		20	55.6%	16	84.2%	4.52, 0.034^∗^
Education, years		8.31 ± 4.49	0.5, 23	8.00 ± 4.03	0, 12	-0.41, 0.680
MMSE (SR: 0–30)		27.03 ± 2.20	20, 30	24.84 ± 3.18	20, 28	-2.53, 0.011^∗^
GDS	1	7	19.4%	4	21.1%	13.40, 0.004^∗∗^
(*n*,%; SR: 1–7)	2	21	58.3%	3	15.8%	
	3	8	22.2%	9	47.4%	
	4	0	0.0%	3	15.8%	
CDR	0	7	19.4%	3	15.8%	0.03, 0.987
(*n*,%; SR: 0–3)	0.5	29	80.6%	14	73.7%	
	1	0	0.0%	2	10.5%	
CDR-SOB	0	7	19.4%	3	15.8%	18.41, 0.018^∗^
(*n*,%; SR: 0–15)	0.5	19	52.8%	3	15.8%	
	1	7	19.4%	5	26.3%	
	1.5	1	2.8%	2	10.5%	
	2	1	0.0%	0	0.0%	
	2.5	0	0.0%	3	15.8%	
	3	1	2.8%	0	0.0%	
	3.5	0	0.0%	2	10.5%	
	4	0	0.0%	1	5.3%	

*p < 0.05, **p < 0.01.^†^ Normal distribution. Data are mean ± SD (minimum–maximum) or number (percent,%). Independent *t* test for continuous variables with normal distribution, Mann–Whitney *U* test for continuous variables without normal distribution, and chi-square test for categorical variables were used. SCD, subjective cognitive decline; MCI, mild cognitive impairment; SD, standard deviation; min, minimum; max, maximum; MMSE, mini-mental state examination; GDS, global deterioration scale; CDR, clinical dementia rating; CDR-SOB, clinical dementia rating-sum of boxes; SR, score range.

### CR, Cognitive Function, and Psychiatric Symptoms

Table 2 shows the results of the CRIq, comprehensive neuropsychological tests, and psychiatric symptom scales. There were no differences of CRIq scores between SCD and MCI groups except for CRI-Working activity that was higher in the SCD group (*Z* = -2.10, *p* = 0.036). The comprehensive neuropsychological test results were generally higher in the SCD group, while psychiatric symptoms were comparable in both groups. Cognitive domain scores in both groups are presented in Supplementary Table S1 in Supplementary Material.

**TABLE 2:** Cognitive reserve, neuropsychological, and psychiatric function of the participants.

	SCD (*n* = 36)		MCI (*n* = 19)		*t* or *Z*, *p*
	Mean ± SD	Min, max	Mean ± SD	Min, max	
**Cognitive reserve index**					
CRI-Education	58.42 ± 12.12	33, 84	58.32 ± 11.64	36, 84	−0.27, 0.790
CRI-Working activity	117.28 ± 15.24	101, 160	110.95 ± 13.31	101, 146	−2.10, 0.036*
CRI-Leisure time^†^	127.19 ± 10.39	107, 144	125.47 ± 7.08	115, 140	0.65, 0.521
CRI-Total^†^	101.22 ± 11.18	78, 124	97.53 ± 10.42	82, 118	1.19, 0.238
**Comprehensive neuropsychological test**					
Digit span forward^†^	−0.06 ± 0.98	−1.59, 2.18	−0.07 ± 1.21	−2.08, 2.24	0.05, 0.958
Digit span backward^†^	−0.30 ± 0.94	−2.06, 1.76	−0.39 ± 1.33	−3.47, 1.91	0.27, 0.789
SVLT, immediate recall^†^	0.47 ± 0.74	−0.84, 1.83	−0.15 ± 1.00	−1.98, 2.07	2.57, 0.013*
SVLT, delayed recall	0.27 ± 1.00	−1.87, 1.87	−0.64 ± 1.29	−2.96, 2.08	−2.72, 0.007*
SVLT, recognition	0.61 ± 0.74	−1.33, 1.65	−0.48 ± 1.75	−3.83, 1.68	−1.99, 0.046*
RCFT, copy	0.13 ± 0.69	−1.45, 1.24	−1.65 ± 2.59	−10.19, 0.49	−3.34, 0.001**
K-BNT ^†^	0.02 ± 0.68	−1.40, 1.05	−1.03 ± 1.26	−2.98, 1.42	4.05, < 0.001***
COWAT, animal ^†^	−0.18 ± 1.06	−2.85, 1.65	−0.76 ± 0.82	−2.36, 0.99	2.09, 0.042*
COWAT, phonemic ^†^	−0.13 ± 0.79	−2.11, 1.20	−0.69 ± 0.82	−1.79, 0.61	2.45, 0.018*
Stroop test, color/word ^†^	0.20 ± 0.85	−1.35, 1.74	−0.42 ± 1.20	−2.83, 1.33	2.24, 0.029*
TMT-A	0.27 ± 0.67	−2.32, 1.12	−2.08 ± 4.43	−14.19, 1.19	−2.75, 0.006**
TMT-B	−0.36 ± 1.25	−4.76, 0.96	−2.60 ± 2.40	−8.49, 0.48	−3.82, <0.001***
**Psychiatric symptoms**					
PANAS-P (SR: 10–50)^†^	19.44 ± 7.19	9, 40	17.68 ± 6.68	11, 34	0.88, 0.380
PANAS-N (SR: 10–50)	19.03 ± 6.42	11, 35	20.53 ± 8.71	12, 39	-0.33, 0.743
K-AES (SR: 18–72)^†^	50.89 ± 10.12	28, 70	47.79 ± 6.86	35, 58	1.20, 0.237
QOL-AD (SR: 13–52)	32.78 ± 7.64	23, 64	31.16 ± 3.85	26, 39	−0.36, 0.723
GDepS (SR: 0–30)^†^	13.86 ± 6.63	0, 29	14.42 ± 7.59	4, 29	−0.28, 0.778

*p < 0.05, **p < 0.01, ***p < 0.001. ^†^ Normal distribution. Independent *t* test for continuous variables with normal distribution and Mann–Whitney *U* test for continuous variables without normal distribution were used. Comprehensive neuropsychological test results are presented as *z*-scores adjusted for age and years of education. SCD, subjective cognitive decline; MCI, mild cognitive impairment; SD, standard deviation; min, minimum; max, maximum; CRI, cognitive reserve index; SVLT, Seoul verbal learning test; RCFT, Rey-Osterrieth complex figure test; K-BNT, Korean version of the Boston naming test; COWAT, controlled oral word association test; TMT, trail making test; PANAS-P, positive and negative affect schedule-positive affect; PANAS-N, positive and negative affect schedule-negative affect; K-AES, Korean version of the apathy evaluation scale; QOL-AD, quality of life-Alzheimer’s disease; GDepS, geriatric depression scale; SR, score range.

### Correlation Between CR and Cognitive Function or Psychiatric Symptoms

Table 3 shows the correlation between CRIq scores and neuropsychological test results and psychiatric symptoms. The MMSE score correlated with CRI-Total (*r* = 0.59, *p* < 0.001), CRI-Education (*r* = 0.54, *p* < 0.001), CRI-Working activity (*r* = 0.39, *p* = 0.003), and CRI-Leisure time (*r* = 0.29, *p* = 0.033) scores. The CDR-SOB score was correlated with CRI-Total (*r* = -0.40, *p* = 0.003), CRI-Education (*r* = -0.29, *p* = 0.031), and CRI-Working activity (*r* = -0.44, *p* = 0.001) scores.

**TABLE 3:** Correlation between cognitive reserve and neuropsychological function and psychiatric symptoms.

	CRI total^†^	CRI education	CRI working activity	CRI leisure time^†^
**Global cognition**				
MMSE	0.59, <0.001***	0.54, <0.001***	0.39, 0.003**	0.29, 0.033*
CDR-SOB	−0.40, 0.003**	−0.29, 0.031*	−0.44, 0.001**	−0.19, 0.157
**Comprehensive neuropsychological test**				
Digit span forward	−0.01, 0.941	−0.02, 0.864	0.00, 0.982	0.02, 0.876
Digit span backward ^†^	−0.13, 0.353	−0.32, 0.015*	−0.06, 0.662	0.03, 0.814
SVLT, immediate recall ^†^	0.22, 0.098	0.12, 0.367	0.04, 0.769	0.30, 0.026*
SVLT, delayed recall	0.23, 0.084	0.20, 0.146	0.15, 0.261	0.27, 0.044*
SVLT, recognition	0.25, 0.066	0.11, 0.435	0.14, 0.323	0.35, 0.009** ^‡^
RCFT, copy	0.21, 0.124	0.01, 0.968	0.18, 0.191	0.38, 0.004** ^‡^
K-BNT ^†^	0.31, 0.019*	0.27, 0.044*	0.19, 0.169	0.35, 0.008** ^‡^
COWAT, animal ^†^	0.03, 0.841	0.06, 0.678	0.02, 0.894	0.08, 0.539
COWAT, phonemic ^†^	0.10, 0.465	−0.06, 0.654	0.05, 0.720	0.35, 0.009** ^‡^
Stroop test, color/word ^†^	0.03, 0.846	−0.01, 0.923	−0.06, 0.671	0.12, 0.398
TMT-A	0.14, 0.294	0.01, 0.933	0.14, 0.294	0.10, 0.464
TMT-B	0.36, 0.006**	0.23, 0.083	0.22, 0.101	0.18, 0.174
**Psychiatric symptoms**				
PANAS-P ^†^	0.23, 0.039*	0.17, 0.205	0.07, 0.634	0.26, 0.052
PANAS-N	0.06, 0.667	−0.00, 0.989	−0.03, 0.856	0.14, 0.294
K-AES ^†^	0.32, 0.018*	0.15, 0.287	0.13, 0.355	0.23, 0.083
QOL-AD	0.22, 0.106	0.13, 0.356	0.04, 0.762	0.24, 0.080
GDepS ^†^	−0.26, 0.052	−0.22, 0.097	−0.19, 0.172	−0.14, 0.317

*p < 0.05, **p < 0.01, ***p < 0.001. ^†^ Normal distribution. ^‡^ Survived *post hoc* tests for multiple comparisons by Benjamini–Hochberg’s method. Data are Pearson’s *r* coefficients (between variables with normal distribution) or Spearman’s rho coefficients (correlation including variables without normal distribution) and *p* values. CRI, cognitive reserve index; MMSE, mini-mental state examination; CDR-SOB, clinical dementia rating-sum of boxes; SVLT, Seoul verbal learning test; RCFT, Rey-Osterrieth complex figure test; K-BNT, Korean version of the Boston naming test; COWAT, controlled oral word association test; TMT, trail making test; PANAS-P, positive and negative affect schedule-positive affect; PANAS-N, positive and negative affect schedule-negative affect; K-AES, Korean version of the apathy evaluation scale; QOL-AD, quality of life-Alzheimer’s disease; GDepS, geriatric depression scale.

After correction for multiple comparisons, the correlation between CRIq scores and some comprehensive neuropsychological test results failed to remain significant, except for the correlation between CRI-Leisure activity with verbal learning test recognition (rho = 0.35, *p* = 0.009), RCFT copy (rho = 0.38, *p* = 0.004), naming ability (*r* = 0.35, *p* = 0.008), and phonemic fluency test (*r* = 0.35, *p* = 0.009). The correlation between CRI-Total score and psychiatric symptoms failed to survive the correction for multiple comparisons. Correlation analyses data for each group are shown in Supplementary Tables S2, S3 in Supplementary Material.

### Regression Analyses on the Association Between CR and Cognitive Functions

**FIGURE 1:**
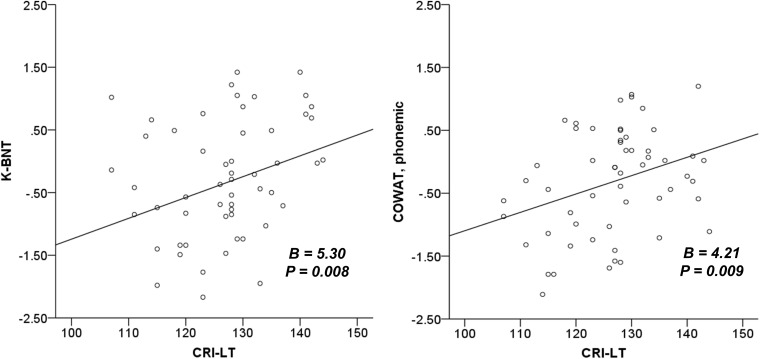
The association between cognitive reserve and global cognition and naming ability. K-BNT is presented as *z*-score adjusted for age and years of education. MMSE, mini-mental state examination; CRI, cognitive reserve index; K-BNT, Korean version of the Boston naming test; CRI-LT, CRI-Leisure time.

The results of our regression analyses revealed the effect of CR on neuropsychological functions that were significant according to our correlation analyses (Table 4). CRI-Total (*B* = 3.00, *p* = 0.005), CRI-Education (*B* = 3.39, *p* = 0.002), and CRI-Leisure time (*B* = 2.56, *p* = 0.015) were prognostic for MMSE after adjusting for sex, diagnostic group, and CDR-SOB. No CR score was prognostic for CDR-SOB after adjusting for sex, diagnostic group, and MMSE. CRI-Leisure time significantly predicted naming ability (*B* = 2.20, *p* = 0.033). Figure 1 shows the association between CRI-Total and MMSE and between CRI-Leisure time and naming ability. Regression analyses data for each group are shown in Supplementary Tables S4, S5 in Supplementary Material.

**TABLE 4:** Multivariable linear regression analysis for cognitive reserve variables in predicting global and detailed neuropsychological function.

Independent variables	B	Standard error	β	*t*, *p*	Model fitness
**Dependent variable: MMSE**					
CRI-Total ^†^	0.004	0.001	0.41	3.00, 0.005**	*F* = 9.63
Sex	0.003	0.03	0.01	0.08, 0.937	*p* < 0.001***
Diagnosis group	−0.07	0.03	−0.28	−2.08, 0.044*	*R*^2^ = 0.49
CDR-SOB	−0.04	0.03	−0.21	−1.46, 0.153	Adj-*R*^2^ = 0.44

CRI-Education	0.22	0.07	0.41	3.39, 0.002**	*F* = 10.65
Sex	−0.01	0.03	−0.04	−0.34, 0.738	*p* < 0.001***
Diagnosis group	−0.08	0.03	−0.33	−2.52, 0.016*	*R*^2^ = 0.52
CDR-SOB	−0.04	0.02	−0.22	−1.54, 0.131	Adj-*R*^2^ = 0.47

CRI-Working activity	0.10	0.17	0.11	0.61, 0.547	*F* = 6.19
Sex	−0.02	0.05	−0.06	−0.34, 0.738	*p* = 0.001**
Diagnosis group	−0.07	0.03	−0.28	−1.89, 0.066	*R*^2^ = 0.38
CDR-SOB	−0.06	0.03	−0.34	−2.12, 0.035*	Adj-*R*^2^ = 0.32

CRI-Leisure time	0.01	0.002	0.35	2.56, 0.015*	*F* = 8.64
Sex	−0.07	0.04	−0.30	−2.10, 0.042*	*p* < 0.001***
Diagnosis group	−0.06	0.03	−0.26	−1.89, 0.066	*R*^2^ = 0.46
CDR-SOB	−0.03	0.03	−0.16	−1.01, 0.320	Adj-*R*^2^ = 0.41

**Dependent variable: CDR-SOB**					
CRI-Total	−0.01	0.01	−0.16	−1.02, 0.312	*F* = 7.68
Sex	0.24	0.20	0.17	1.22, 0.231	*p* < 0.001***
Diagnosis group	0.42	0.19	0.30	2.18, 0.035*	*R*^2^ = 0.43
MMSE	−1.39	0.95	−0.34	−1.46, 0.153	Adj-*R*^2^ = 0.38

CRI-Education	−0.32	0.48	−0.10	−0.68, 0.500	*F* = 7.436
Sex	0.29	0.19	0.21	1.55, 0.130	*p* < 0.001***
Diagnosis group	0.44	0.20	0.32	2.20, 0.034*	*R*^2^ = 0.43
MMSE	−1.51	0.98	−0.26	−1.54, 0.131	Adj-*R*^2^ = 0.37

CRI-Working activity	0.02	0.96	0.004	0.02, 0.982	*F* = 7.23
Sex	0.32	0.25	0.23	1.28, 0.206	*p* < 0.001***
Diagnosis group	0.40	0.19	0.29	2.08, 0.044*	*R*^2^ = 0.42
MMSE	−1.86	0.85	−0.32	−2.19, 0.035*	Adj-*R*^2^ = 0.36

**Dependent variable: SVLT recognition**					
CRI-Leisure time	0.01	0.02	0.06	0.26, 0.797	*F* = 1.36
Sex	0.49	0.40	0.28	1.21, 0.235	*p* = 0.275
Diagnosis group	−0.28	0.37	−0.14	−0.75, 0.459	*R*^2^ = 0.17
CDR-SOB	−0.54	0.37	−0.31	−1.47, 0.152	Adj-*R*^2^ = 0.04

**Dependent variable: RCFT copy**					
CRI-Leisure time ^†^	−0.03	0.03	−0.25	−0.83, 0.419	*F* = 0.67
Sex	−0.09	0.52	−0.05	−0.17, 0.870	*p* = 0.623
Diagnosis group	−0.66	0.58	−0.33	−1.15, 0.267	*R*^2^ = 0.14
CDR-SOB	−0.16	0.45	−0.12	−0.35, 0.730	Adj-*R*^2^ = -0.07

**Dependent variable: K-BNT**^†^					
CRI-Leisure time ^†^	0.04	0.02	0.33	2.20, 0.033*	*F* = 6.10
Sex	−0.15	0.35	−0.07	−0.44, 0.662	*p* = 0.001**
Diagnosis group	−0.98	0.32	−0.45	−3.06, 0.004**	*R*^2^ = 0.38
CDR-SOB	−0.001	0.27	−0.001	−0.01, 0.996	Adj-*R*^2^ = 0.32

**Dependent variable: COWAT phonemic**^†^					
CRI-Leisure time ^†^	0.01	0.02	0.13	0.77, 0.448	*F* = 1.67
Sex	−0.08	0.32	−0.04	−0.24, 0.811	*p* = 0.176
Diagnosis group	−0.39	0.29	−0.23	−1.35, 0.186	*R*^2^ = 0.14
CDR-SOB	−0.14	0.25	−0.11	−0.56, 0.581	Adj-*R*^2^ = 0.06

*p < 0.05, **p < 0.01, ***p < 0.001. ^†^ Normal distribution. For each dependent variable, multivariable regression analysis was performed. Log transformation was performed for variables without normal distribution. Sex was coded as 1 (male) or 2 (female), and diagnostic group was coded as 0 (SCD) or 1 (MCI). MMSE, mini-mental state examination; CRI, cognitive reserve index; CDR-SOB, clinical dementia rating-sum of boxes; Adj-*R*^2^, adjusted *R*^2^; SVLT, Seoul verbal learning test; RCFT, Rey-Osterrieth complex figure test; K-BNT, Korean version of the Boston naming test; COWAT, controlled oral word association test; SCD, subjective cognitive decline; MCI, mild cognitive impairment.

### Multivariable Regression Model

We used a multivariable regression model to test the relationship among CRI-Education, CRI-Working activity, CRI-Leisure time, and the composite cognitive score calculated from the comprehensive neuropsychological test. The results were then presented as a path diagram. The association between education and occupation is well known (Ng and Feldman, 2009; Nucci et al., 2012; Choi et al., 2016). Hence, we hypothesized the correlation between education and working activity and added the covariance between CRI-Education and CRI-Working activity in the model, which showed an adequate fit (χ^2^ = 2.45, RMSEA = 0.064, CFI = 0.973).

**FIGURE 2:**
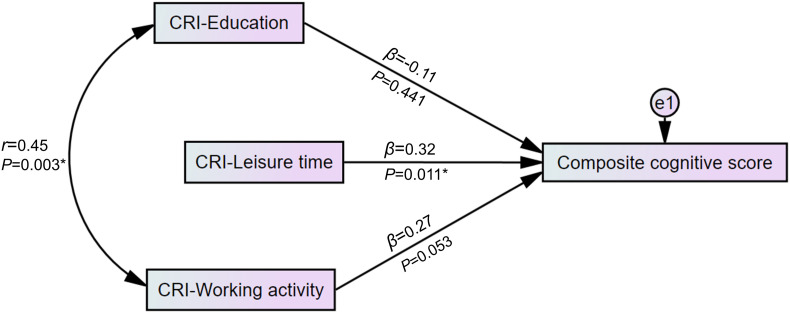
Path diagram of the association between the domains of cognitive reserve and the composite cognitive score. Standardized scores of detailed neuropsychological tests were used to construct a composite cognitive score. In a multivariable regression model with an adequate fit (χ^2^ = 2.45, RMSEA = 0.064, CFI = 0.973), CRI-Leisure time showed a significant regression weight for the composite cognitive score. *p < 0.05. CRI, cognitive reserve index; e1, residual error variable; β, standardized regression weight; *r*, correlation estimate; RMSEA, root-mean-square error of approximation; CFI, Comparative Fit Index.

Figure 2 graphically displays a significant regression estimate of CRI-Leisure time on the composite cognitive score (β = 0.32, *p* = 0.011). CRI-Education (β = -0.11, *p* = 0.441) and CRI-Working activity (β = 0.27, *p* = 0.053) did not show significant regression estimates on the composite cognitive score. Correlation between CRI-Education and CRI-Working activity (*r* = 0.45, *p* = 0.003 as shown in Figure 2) was found.

We also used a multivariable regression model to determine the association between CRI subdomains and cognitive subdomains. The result shows that CR-Leisure activity is predictive of cognitive subdomains such as language function (β = 0.29, *p* = 0.021), memory (β = 0.33, *p* = 0.009), visuospatial function (β = 0.29, *p* = 0.019), and frontal executive function (β = 0.26, *p* = 0.041), while CRI-Working activity predicts visuospatial function (β = 0.33, *p* = 0.015). CRI-Education failed to predict any cognitive domain. The results are presented in Supplementary Figure S1 in the Supplementary Material.

## Discussion

In the present study, we hypothesized that the lifetime comprehensive CR may positively associate with neuropsychological function and psychiatric symptoms in patients at the early stage of cognitive decline. The results showed that total CR and its subdomains of education, working activity, and leisure time were associated with global cognitive function. In addition, only the CR based on leisure activity was associated with naming ability and the composite cognitive score in the early stage of cognitive decline, while education and working activity showed no association with detailed neuropsychological function and composite cognitive score.

As expected, we found a correlation between comprehensive CR and global cognition in patients at the early stage of cognitive decline. In the correlation analyses, the MMSE score, which reflects global cognition, was correlated with all CR proxies, including total CR, education, working activity, and leisure time scores, and its association with total CR, education, and leisure time remained significant in the regression analyses. These results are in line with previous research findings that a higher CR is associated with a higher cognition in global measures in healthy older adults (Liu et al., 2013; Lavrencic et al., 2018). Since old individuals with a high CR can respond flexibly to the beginning of cognitive decline due to aging or neurodegeneration (Scarmeas, 2007), our results in patients with SCD or MCI can support the CR theory that a higher CR, accumulated through a lifetime of brain stimulating activities, increases cognitive plasticity and delays the onset of MCI or dementia (Stern, 2002, 2012; Liu et al., 2013).

The associations between the subdomain scores of CR and the detailed neuropsychological tests representing the five cognitive domains are the main results of the present study. In the correlation analyses, the CRI-Total score correlated with the naming ability and executive function, which corresponds with previous results that a high level of CR was associated with the naming ability and divided attention (Puccioni and Vallesi, 2012; Cabral et al., 2016). We also found a correlation between educational attainment and naming ability. However, the effect of total CR and education disappeared after correction for multiple comparisons. It is well-established that there is a positive association between education and various cognitive functions, including memory, attention, and executive function in healthy older adults (Cabral et al., 2016; Opdebeeck et al., 2016; Zhang et al., 2019). However, other studies have also reported that education is associated with the level of cognitive performance in healthy older adults but not with the rate of cognitive decline itself (Bendayan et al., 2017; Seblova et al., 2020). Our result also showed little association between working activity and detailed neuropsychological tests in both correlation and regression analyses, implying little effect of working activities on detailed cognition at the early stage of cognitive decline. Previous studies have reported that although occupational attainment was a protective factor for cognitive decline in healthy older adults (Smart et al., 2014; Boots et al., 2015), it was a risk factor for progression from MCI to AD (Myung et al., 2017). Similarly, a large longitudinal study has suggested a paradoxical relationship where CR is associated with slower cognitive decline in normal old adults and MCI due to AD patients and rapid decline in AD dementia patients (van Loenhoud et al., 2019). We assume that the difference we observed in the association between subdomains of CR and detailed neuropsychological function might be attributable to the characteristics of our participants who were at the early stage of cognitive decline.

Another main finding is the association between leisure activity and detailed neuropsychological functions. The regression analyses revealed that only lifetime leisure activity was associated with preserved naming ability. Moreover, in the competitive multivariable regression model, the composite cognitive score based on the 12 neuropsychological subtests and the cognitive domain scores based on language function, memory, visuospatial function, and frontal executive function were explained by leisure activity, and not by education and working activity. The measures for CR used in previous studies have also included leisure activities, such as reading books, newspapers, magazines (Leon et al., 2014; Cabral et al., 2016), playing games, playing a musical instrument, or collecting things (Leon et al., 2014). CR could reportedly enhance verbal memory, executive function, and attention in healthy older adults (Puccioni and Vallesi, 2012; Cabral et al., 2016). In our study, we used CRIq, which thoroughly measures lifetime leisure activity, and includes weekly activities, such as reading newspapers and magazines, sports, driving, dancing, and using new technologies; monthly activities, such as social gathering, going to cinema or theater, gardening, handicraft, taking care of children or elderly people, and playing instruments; and annual activities, such as watching concerts, attending conferences, going on overnight trips, or the number of books read (Nucci et al., 2012). We believe that this comprehensive evaluation of lifetime leisure activity may have affected the significant relationship between CRI-Leisure time scores and cognitive function in naming ability and cognitive domain scores even in the beginning of cognitive decline. Leisure activities were consistently found to be protective against cognitive decline and incident dementia in healthy older adults (Akbaraly et al., 2009; Wang et al., 2012). In addition, the participants in our study were in the early stage of cognitive decline and CR might have been utilized as a compensatory mechanism in the aspects of everyday life (Tomaszewski Farias et al., 2018) and neural networks (Liang et al., 2011) against cognitive decline (Stern, 2012). The low education level in our study also may have contributed to the effect of leisure on cognitive function, because education has been reported to affect the association between leisure activity and cognitive function in a previous study, where an association between leisure activities and cognitive function was only observed in low-educated old adults (Park et al., 2019). We assume that lifetime leisure activities can help adapt to the early cognitive decline, particularly in naming ability and cognitive domains including language, memory, visuospatial, and frontal executive function through abstract and mental stimulating activities.

An association between CR and psychiatric symptoms was not found in our study. We assessed psychiatric symptoms that start to become more frequent in the early stage of dementia (Lyketsos et al., 2011), but failed to find any association between CR and apathy, affect, QOL, and depression. Apathy and a low positive affect are known to be the main behavioral and psychological symptoms of dementia commonly observed in various types of dementia (Gilley et al., 1991; Harrison et al., 2016; Breitve et al., 2018). Lower mood, motivation, activity, and affect can be manifested as the prodromal or initial symptoms in the dementia continuum (van Dalen et al., 2018; Tay et al., 2020). Apathy is also known to persist during the course of the disease (van der Linde et al., 2016) and is highly associated with impaired cognitive function (Reichman et al., 1996; Brown and Pluck, 2000; Breitve et al., 2018). In this context, a highly accumulated CR may help maintain the motivation and positive affect through various experiences and learnings over a lifetime at the very beginning of dementia. However, CR did not show a correlation with apathy and positive effect in this study after correction of multiple comparisons for psychiatric symptoms. This inconsistency may be attributed to the small size of our study leading to a low statistical power. Larger studies in the future may find significant associations.

We note several strengths and limitations to our study. The strength of the present study is in the characteristics of the participants who were at the early stage of cognitive decline and the measurement of CR and neuropsychological function. The most comprehensive validated measure for CR (Kartschmit et al., 2019) and the five domains of cognition evaluated in our study enabled us to determine that only leisure activity predicted cognitive decline in participants in early stage of cognitive decline. Popular and well-validated screening tests including functional ability and psychiatric symptoms are also other strengths of our study. However, the small sample size and low education level in our participants can limit the generalizability of our results. In addition, although we initially aimed to recruit participants at the pre-dementia stage, the sample was relatively heterogeneous, including those who were diagnosed with MCI and SCD. Due to the lack of information on AD biomarker evaluation, the type of dementia, i.e., AD, vascular dementia, dementia of Lewy body, and subcortical vascular dementia, was also not determined. Although SCD can be a potential risk factor for MCI or dementia, the majority of individuals with SCD were cognitively stable in longitudinal studies (Hessen et al., 2017; Lee et al., 2020). In addition, the neuropsychological symptoms of dementia also differ based on the type of dementia. Hence, the participants in our study can represent various neuropsychological profiles, which can be another limitation. Despite the lack of dementia biomarker evaluation, such as brain imaging or a CSF study, experienced clinicians in our study confirmed the diagnosis clinically based on the DSM-5 (American Psychiatric Association, 2013), Petersons’ criteria (Petersen, 2004), and research criteria of SCD (Jessen et al., 2014) and effectively excluded participants with dementia; other major psychiatric disorders, such as major depressive disorder and anxiety disorders; and neurologic diseases, such as Parkinson’s disease, epilepsy, and cerebrovascular disease.

In conclusion, we found an association between comprehensive CR and global cognition in patients at the early stage of cognitive decline. In particular, the lifetime leisure activity predicted naming ability and cognitive functions in domains including language, memory, visuospatial, and frontal executive function. These results may highlight the mental and social stimulation of leisure activity on maintaining cognition levels at the beginning of cognitive decline. Future studies should include a large number of participants with normal cognition, SCD, MCI, and dementia to compare the association between comprehensive CR and cognition across the diagnostic groups in the continuum of dementia. Additional evaluation of the biomarkers of cognitive decline would identify the neural mechanism of CR in aging and neurodegeneration.

## Data Availability Statement

The datasets generated for this study are available on request from the corresponding author.

## Ethics Statement

The studies involving human participants were reviewed and approved by the Institutional Review Board of Gachon University Gil Medical Center (Approval number: GAIRB2019-230). The participants provided written informed consent to participate in this study.

## Author Contributions

S-JC, J-YL, and JK designed the study. SL, JK, DK, SW, and S-JC acquired the data. SL, JK, and DK analyzed the data. SL and JK wrote the manuscript. SW, J-YL, and S-JC critically reviewed the manuscript for important intellectual content. All authors reviewed and approved the final version of the manuscript for publication.

## Conflict of Interest

The authors declare that the research was conducted in the absence of any commercial or financial relationships that could be construed as a potential conflict of interest.

## Abbreviations

AD: Alzheimer‗s disease
AES: apathy evaluation scale
CDR-SOB: clinical dementia rating-sum of boxes
COWAT: controlled oral word association test
CR: cognitive reserve
CRI: cognitive reserve index
CRIq: cognitive reserve index questionnaire
GDepS: geriatric depression scale
GDS: global deterioration scale
IQ: intelligence quotient
K-BNT: Korean version of the Boston naming
MMSE: mini-mental state examination
MCI: mild cognitive impairment
PANAS: positive and negative affect schedule
RCFT: Rey-Osterrieth complex figure test
RMSEA: root-mean-square error of approximation
SCD: subjective cognitive decline
SCLT: Seoul verbal learning test
TMT Ü A: trail making test-A.
