# Supplementary material for: Cognitive Reserve, Leisure Activity, and Neuropsychological Profile in the Early Stage of Cognitive Decline
Source: Front Aging Neurosci. 2020 Oct 26;12:590607. doi: 10.3389/fnagi.2020.590607 (PMC7649371; doi:10.3389/fnagi.2020.590607)

**Table S1.** Comparison of the cognitive domain scores between SCD and MCI groups

| **Cognitive domain** | **Mean, SD** | | **t, p value** |
| --- | --- | --- | --- |
|  | **SCD (n=36)** | **MCI (n=19)** |  |
| Attention | -0.03, 0.61 | -0.85, 1.91 | 1.82, 0.084 |
| Language | 0.02, 0.68 | -1.03, 1.26 | 3.40, 0.002^*^ |
| Memory | 0.45, 0.73 | -0.42, 1.13 | 3.49, 0.001^*^ |
| Visuospatial function | 0.13, 0.69 | -1.65, 2.59 | 2.94, 0.008^*^ |
| Frontal executive function | -0.12, 0.60 | -1.12, 1.04 | 3.87, 0.001^*^ |
| ^*^*p*<0.05  All scores distributed normally. Independent t-test was u  Comprehensive neuropsychological test results are presented as z-scores adjusted for age and years of education.  Attention: digit span forward, digit span backward, trail making test-A  Language: Korean version of Boston naming test  Memory: Seoul verbal learning test immediate recall/delayed recall/recognition  Visuospatial function: Rey-Osterrieth complex figure test, copy  Frontal executive function: world fluency test animal/phonemic, stroop color/word reading test, trail making test-B  Abbreviations: SD = standard deviation; SCD = subjective cognitive decline; MCI = mild cognitive impairment | | | |

**Table S2.** Correlation between cognitive reserve and neuropsychological function and psychiatric symptoms in SCD group (n=36)

|  | | | | |
| --- | --- | --- | --- | --- |
|  | **CRI total** ^†^ | **CRI education** | **CRI working activity** | **CRI leisure time** |
| **Global cognition** | | | | |
| MMSE ^†^ | 0.56, <0.001^***^ | 0.61, <0.001^***^ | 0.28, 0.105 | 0.23, 0.170 |
| CDR-SOB | -0.20, 0.234 | -0.17, 0.325 | -0.39, 0.020^*^ | 0.02, 0.919 |
| **Comprehensive neuropsychological test** | | | | |
| Digit span forward ^†^ | 0.04, 0.813 | -0.10, 0.958 | -0.05, 0.759 | 0.18, 0.288 |
| Digit span backward ^†^ | -0.28, 0.097 | -0.36, 0.030^*, ‡^ | -0.14, 0.427 | -0.07, 0.702 |
| SVLT, immediate recall ^†^ | 0.12, 0.496 | 0.18, 0.289 | -0.16, 0.368 | 0.30, 0.080 |
| SVLT, delayed recall ^†^ | -0.08, 0.649 | 0.20, 0.246 | -0.15, 0.373 | 0.19, 0.273 |
| SVLT, recognition | 0.10, 0.573 | 0.13, 0.440 | -0.11, 0.534 | 0.23, 0.185 |
| RCFT, copy ^†^ | 0.20, 0.241 | 0.08, 0.665 | -0.00, 0.994 | 0.40, 0.017^*, ‡^ |
| K-BNT ^†^ | 0.11, 0.520 | 0.11, 0.529 | -0.06, 0.740 | 0.23, 0.186 |
| COWAT, animal ^†^ | -0.05, 0.759 | 0.01, 0.978 | -0.10, 0.583 | 0.03, 0.882 |
| COWAT, phonemic ^†^ | 0.00, 0.993 | -0.10, 0.547 | -0.09, 0.592 | 0.26, 0.122 |
| Stroop test, color reading ^†^ | -0.08, 0.636 | -0.03, 0.850 | -0.17, 0.327 | 0.08, 0.654 |
| TMT-A ^†^ | -0.12, 0.506 | -0.06, 0.699 | -0.10, 0.575 | -0.07, 0.699 |
| TMT-B | -0.07, 0.771 | -0.24, 0.342 | 0.18, 0.486 | -0.31, 0.216 |
| **Psychiatric symptoms** | | | | |
| PANAS-P ^†^ | 0.41, 0.012^*, ‡^ | 0.32, 0.057 | 0.22, 0.205 | 0.31, 0.069 |
| PANAS-N ^†^ | 0.00, 0.999 | 0.06, 0.744 | -0.12, 0.474 | 0.11, 0.513 |
| K-AES ^†^ | 0.40, 0.016^*, ‡^ | 0.20, 0.233 | 0.27, 0.115 | 0.32, 0.061 |
| QOL-AD | 0.22, 0.201 | 0.11, 0.515 | 0.04, 0.835 | 0.33, 0.051 |
| GDepS ^†^ | -0.38, 0.023^*, ‡^ | -0.22, 0.191 | -0.40, 0.015^*, ‡^ | -0.04, 0.819 |
| ^*^*p*<0.05, ^**^*p*<0.01, ^***^*p*<0.001  ^†^ Normally distributed variables  ^‡^ Insignificant after post-hoc tests for multiple comparisons by Benjamini Hochberg’s method.  All significant variables in comprehensive neuropsychological test and psychiatric symptoms did not survive post-hoc tests for multiple comparisons by Benjamini Hochberg’s method.  Data are Pearson’s r coefficients (between variables with normal distribution) or Spearman’s rho coefficients (correlation including variables without normal distribution) and *p* values.  Abbreviations: CRI = cognitive reserve index; MMSE = mini-mental state examination; CDR-SOB = clinical dementia rating–sum of boxes SVLT = Seoul verbal learning test; RCFT = Rey-Osterrieth complex figure test; K-BNT = Korean version of the Boston naming test; COWAT = controlled oral word association test; TMT= trail making test; PANAS-P = positive and negative affect schedule-positive affect; PANAS-N = positive and negative affect schedule-negative affect; K-AES = Korean version of the apathy evaluation scale; QOL-AD = quality of life-Alzheimer's disease; GDepS = geriatric depression scale. | | | | |

**Table S3.** Correlation between cognitive reserve and neuropsychological function and psychiatric symptoms in MCI group (n=19)

|  | | | | |
| --- | --- | --- | --- | --- |
|  | **CRI total** ^†^ | **CRI education** | **CRI working activity** | **CRI leisure time** ^†^ |
| **Global cognition** | | | | |
| MMSE ^†^ | 0.63, 0.004^**^ | 0.50, 0.030^*^ | 0.40, 0.088 | 0.53, 0.020^*^ |
| CDR-SOB | -0.44, 0.061 | -0.14, 0.556 | -0.36, 0.132 | -0.60, 0.007^*^ |
| **Comprehensive neuropsychological test** | | | | |
| Digit span forward^†^ | -0.11, 0.651 | -0.07, 0.779 | 0.05, 0.831 | -0.32, 0.186 |
| Digit span backward ^†^ | 0.07, 0.782 | -0.05, 0.839 | 0.04, 0.888 | 0.15, 0.532 |
| SVLT, immediate recall ^†^ | 0.30, 0.212 | 0.21, 0.931 | 0.34, 0.150 | 0.19, 0.444 |
| SVLT, delayed recall^†^ | 0.33, 0.165 | 0.11, 0.965 | 0.36, 0.130 | 0.31, 0.203 |
| SVLT, recognition ^†^ | 0.27, 0.256 | 0.01, 0.959 | 0.28, 0.253 | 0.45, 0.054 |
| RCFT, copy | 0.11, 0.653 | -0.19, 0.446 | 0.24, 0.324 | 0.27, 0.268 |
| K-BNT ^†^ | 0.50, 0.030^*, ‡^ | 0.56, 0.013^*, ‡^ | 0.12, 0.625 | 0.50, 0.031^*, ‡^ |
| COWAT, animal ^†^ | 0.08, 0.732 | -0.03, 0.891 | 0.02, 0.936 | 0.24, 0.331 |
| COWAT, phonemic ^†^ | 0.15, 0.543 | 0.02, 0.940 | -0.00, 0.986 | 0.50, 0.030^*, ‡^ |
| Stroop test, color reading ^†^ | 0.06, 0.794 | 0.00, 0.999 | 0.01, 0.963 | 0.06, 0.823 |
| TMT-A | 0.21, 0.660 | 0.68, 0.095 | -0.20, 0.661 | 0.36, 0.432 |
| TMT-B ^†^ | 0.09, 0.721 | -0.06, 0.815 | 0.09, 0.730 | 0.27, 0.272 |
| **Psychiatric symptoms** | | | | |
| PANAS-P ^†^ | -0.08, 0.746 | -0.15, 0.545 | -0.06, 0.797 | 0.13, 0.593 |
| PANAS-N ^†^ | 0.09, 0.709 | -0.14, 0.564 | 0.21, 0.385 | 0.19, 0.426 |
| K-AES ^†^ | 0.01, 0.970 | 0.01, 0.979 | 0.01, 0.971 | -0.02, 0.924 |
| QOL-AD ^†^ | -0.03, 0.894 | 0.03, 0.915 | -0.12, 0.618 | 0.09, 0.726 |
| GDepS | -0.06, 0.821 | -0.26, 0.292 | 0.23, 0.340 | -0.22, 0.375 |
| ^*^*p*<0.05, ^**^*p*<0.01  ^†^ Normally distributed variables  ^‡^ Insignificant after post-hoc tests for multiple comparisons by Benjamini Hochberg’s method.  All significant variables in comprehensive neuropsychological test and psychiatric symptoms did not survive post-hoc tests for multiple comparisons by Benjamini Hochberg’s method.  Data are Pearson’s r coefficients (between variables with normal distribution) or Spearman’s rho coefficients (correlation including variables without normal distribution) and *p* values.  Abbreviations: CRI = cognitive reserve index; MMSE = mini-mental state examination; CDR-SOB = clinical dementia rating–sum of boxes SVLT = Seoul verbal learning test; RCFT = Rey-Osterrieth complex figure test; K-BNT = Korean version of the Boston naming test; COWAT = controlled oral word association test; TMT= trail making test; PANAS-P = positive and negative affect schedule-positive affect; PANAS-N = positive and negative affect schedule-negative affect; K-AES = Korean version of the apathy evaluation scale; QOL-AD = quality of life-Alzheimer's disease; GDepS = geriatric depression scale. | | | | |

**Table S4.** Multivariable linear regression analysis results for cognitive reserve variables predicting global and detailed neuropsychological test results in SCD group

|  | | | | | | |
| --- | --- | --- | --- | --- | --- | --- |
| **Independent variables** | | **B** | **standard**  **error** | **Β** | **t, p** | **Model fitness** |
| **Dependent variable: MMSE** ^†^ | | | |  |  |  |
| CRI-Total ^†^ | 0.10 | | 0.04 | 0.49 | 2.74, 0.011^*^ | F = 5.75  p = 0. 004^**^  R^2^ = 0.41  Adj-R^2^ = 0.34 |
| Sex | 0.40 | | 0.80 | 0.08 | 0.49, 0.627 |  |
| CDR-SOB | -1.38 | | 0.84 | -0.29 | -1.64, 0.113 |  |
| CRI-Education ^†^ | 7.29 | | 1.95 | 0.66 | 3.75, 0.001^**^ | F = 8.59  p < 0. 001^***^  R^2^ = 0.51  Adj-R^2^ = 0.45 |
| Sex | 0.14 | | 0.71 | 0.03 | 0.20, 0.846 |  |
| CDR-SOB | -0.50 | | 0.85 | -0.11 | -0.59, 0.561 |  |
| **Dependent variable: CDR-SOB** | | |  |  |  |  |
| CRI-Working activity | 0.18 | | 0.93 | 0.05 | 0.19, 0.849 | F = 3.46  p = 0. 031^*^  R^2^ = 0.29  Adj-R^2^ = 0.21 |
| Sex | 0.28 | | 0.23 | 0.29 | 1.22, 0.234 |  |
| MMSE | -0.10 | | 0.04 | -0.43 | -2.46, 0.021^*^ |  |
| ^*^*p*<0.05, ^**^*p*<0.01, ^***^*p*<0.001, ^†^ Normal distribution  For each dependent variable, multivariable regression analysis was performed. Log-transformation was performed for variables without normal distribution. Sex was coded as 1 (male) or 2 (female) and diagnostic group was coded as 0 (SCD) or 1 (MCI).  Abbreviations: MMSE = mini-mental state examination; CRI = cognitive reserve index; CDR-SOB = clinical dementia rating–sum of boxes; Adj-R^2^ = adjusted R^2^; RCFT = Rey-Osterrieth complex figure test; PANAS-P = positive and negative affect schedule-positive affect; K-AES = Korean version of the apathy evaluation scale; GDepS = geriatric depression scale; SCD = subjective cognitive decline; MCI = mild cognitive impairment | | | | | | |

**Table S5.** Multivariable linear regression analysis results for cognitive reserve variables predicting global and detailed neuropsychological test results in MCI group

|  | | | | | | |
| --- | --- | --- | --- | --- | --- | --- |
| **Independent variables** | | **B** | **standard**  **error** | **Β** | **t, p** | **Model fitness** |
| **Dependent variable: MMSE** ^†^ | | | |  |  |  |
| CRI-Total ^†^ | 0.10 | | 0.10 | 0.33 | 0.99, 0.344 | F = 1.43  p = 0.282  R^2^ = 0.26  Adj-R^2^ = 0.08 |
| Sex | -1.41 | | 2.67 | -0.18 | -0.53, 0.608 |  |
| CDR-SOB | -0.38 | | 1.03 | -0.10 | -0.37, 0.717 |  |
| **Dependent variable: MMSE** ^†^ | | | |  |  |  |
| CRI-Education | | 3.09 | 3.58 | 0.23 | 0.86 0.405 | F = 1.34  p = 0.309  R^2^ = 0.25  Adj-R^2^ = 0.06 |
| Sex | | -2.29 | 2.30 | -0.29 | -1.00, 0.338 |  |
| CDR-SOB | | -0.66 | 1.04 | -0.17 | -0.64, 0.537 |  |
| **Dependent variable: MMSE** ^†^ | |  |  |  |  |  |
| CRI-Leisure time ^†^ | | 0.22 | 0.13 | 0.53 | 1.66, 0.122 | F = 2.18  p = 0.143  R^2^ = 0.35  Adj-R^2^ = 0.19 |
| Sex | | -2.46 | 2.01 | -0.31 | -1.22, 0.245 |  |
| CDR-SOB | | 0.76 | 1.23 | 0.20 | 0.61, 0.551 |  |
| **Dependent variable: CDR-SOB** | | |  |  |  |  |
| CRI-Leisure time ^†^ | | -0.07 | 0.03 | -0.69 | -2.85, 0.015^*^ | F = 3.96  p = 0.036^*^  R^2^ = 0.50  Adj-R^2^ = 0.37 |
| Sex | | 0.39 | 0.48 | 0.19 | 0.82, 0.431 |  |
| MMSE ^†^ | | 0.04 | 0.07 | 0.15 | 0.61, 0.551 |  |
| ^*^*p*<0.05, ^**^*p*<0.01, ^***^*p*<0.001, ^†^ Normal distribution  For each dependent variable, multivariable regression analysis was performed. Log-transformation was performed for variables without normal distribution. Sex was coded as 1 (male) or 2 (female) and diagnostic group was coded as 0 (SCD) or 1 (MCI).  Abbreviations: MMSE = mini-mental state examination; CRI = cognitive reserve index; CDR-SOB = clinical dementia rating–sum of boxes; Adj-R^2^ = adjusted R^2^; K-BNT = Korean version of the Boston naming test; SCD = subjective cognitive decline; MCI = mild cognitive impairment | | | | | | |

**Figure S1**. Path diagram of multivariable regression model testing association between CRI subdomains and neuropsychological test subdomains


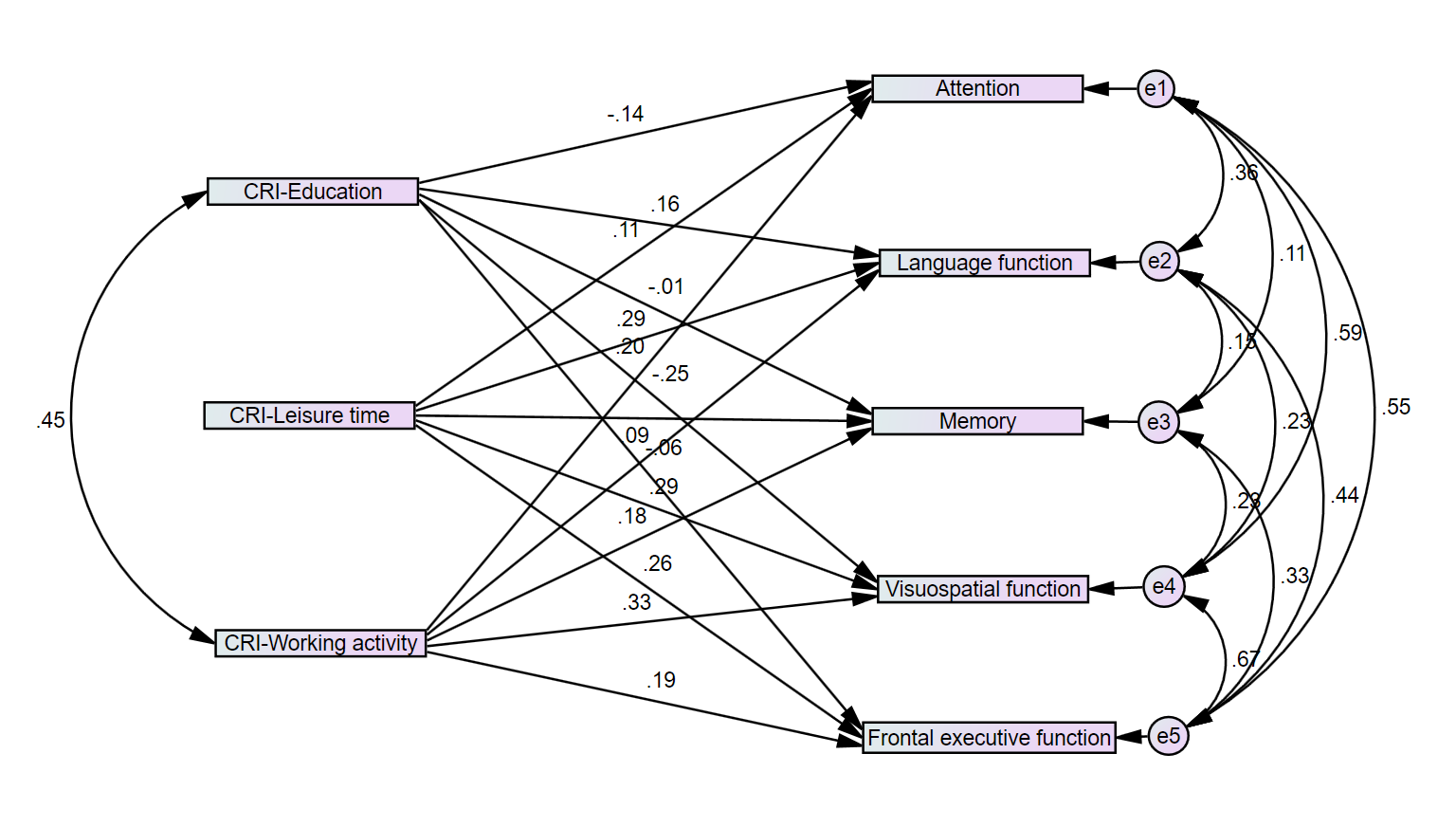

Supplement: Supplementary file 1 [file Data_Sheet_1.DOCX]
